# Optogenetic Analysis of Behavior in the Mosquito *Aedes aegypti*

**DOI:** 10.64898/2026.03.15.711871

**Published:** 2026-03-18

**Authors:** Spruha Rami, Michelle So, Cassandra Travis, Yaoyu Jiao, Paul Shamble, Trevor R. Sorrells

**Affiliations:** 1 Department of Genetics, Yale University, New Haven CT 06510, USA; 2 Howard Hughes Medical Institute, New York, NY 10065, USA; 3 Kavli Institute for Neuroscience, Yale University, New Haven, CT 06510, USA.; 4 Wu Tsai Institute, Yale University, New Haven, CT 06510, USA

## Abstract

The mosquito *Aedes aegypti* is an important vector of viral pathogens and serves as a model for other vector species. Pathogens are transmitted when a mosquito bites a host animal, but the neural circuits that control seeking and biting behavior are not known. Here, we detail methods and protocols for the manipulation of neural activity in the mosquito using optogenetics, a key technique to determine the causal relationship between neural circuits and behavior. These methods include rearing mosquitoes for optogenetics and three assays that are designed to measure different steps in the sequence of arousal, attraction, proboscis probing, and engorgement on the blood of host animals. These behaviors occur at different spatial scales and in response to different sensory stimuli. Each behavioral assay is outfitted with red (625 nm) LEDs for optogenetic activation. To detect arousal in response to olfactory stimuli, flight and walking are measured in all three assays. To assay attraction or landing, mosquitoes are presented with a heated blood meal in a large arena. Proboscis probing and engorgement are assayed with video resolution that enables measurement of appendages and abdomen size. The protocol describes machine vision models to enable high-resolution temporal quantification of behavior as well as endpoint measurements of feeding. These methods can be used to test the function of any population of neurons in mosquito biting behavior and can be extended to additional behaviors.

## Introduction

The mosquito is one of the most dangerous animals in the world, not for its ferocity or strength but for its efficiency as a vector for disease. Malaria-causing parasites spread by *Anopheline* mosquitoes are responsible for hundreds of thousands of deaths yearly, while *Aedes aegypti* and *Aedes albopictus* are responsible for the spread of yellow fever, dengue, chikungunya, Zika fever, and many other life-threatening diseases^1^. Mosquitoes are present on every continent except Antarctica and pose a great concern for public health^2^. Blood-feeding behavior directly transmits pathogens, but it is also an essential step in mosquito life cycle, as a vertebrate blood meal is required to lay eggs. Curbing the spread of mosquito-borne diseases thus requires understanding their host-seeking mechanisms.

It is well-established that female mosquitoes make use of long- and short-distance cues to locate hosts^3,4^. Carbon dioxide (CO_2_) is a highly volatile gas present in human breath that is detected in *Aedes aegypti* olfactory sensory neurons by Gr1, Gr2, and Gr3 proteins, which form the CO_2_ receptor^5^. Exposure to CO_2_ causes take-off and flight behavior, also known as activation^6,7^, which can persist for up to 10–15 minutes, even in the absence of further stimuli^8^. CO_2_ synergizes with skin odor to enhance activation and attraction^5,9^. Following CO_2_ and skin odor detection, the mosquito is primed to sense additional, more proximal host cues, such as heat, visual contrast, and humidity that guide her to land on a viable blood meal host^5,10–13^. Integration of multiple sensory cues allows mosquitoes to effectively pursue and locate their host.

When a female mosquito lands on a potential host, she thrusts her proboscis into the surface until a patch of skin is located, a process known as probing^14,15^. Following probing, her stylet pierces the skin, locates a blood vessel, and simultaneously ingests blood while injecting saliva, doubling her body weight in several minutes^16^. Blood is directed to the midgut, where proteins are digested for egg production. Interestingly, engorgement on a host is not necessary for pathogen spread, as probing alone can transmit disease^16,17^. Therefore, understanding each of the steps in biting behavior could allow the development of strategies to disrupt female mosquito host seeking and biting and ultimately to control the spread of mosquitoborne diseases.

Numerous tools have been created in model organisms for manipulating neuronal activity. Early tools worked constitutively or by thermal activation^18–21^. While useful, these genetic reagents pose certain drawbacks. Constitutive tools can affect development, leading to phenotypes not due solely to the circuit function of the neurons. Mosquitoes use body heat to identify host vertebrates, making thermally activated channels uniquely unsuitable for studies of their behavior^22,23^.

In studies of behavior, optogenetics refers to the use of genetic tools that respond to specific wavelengths of light to control the behavior of neurons of interest. This technique has already been used in multiple systems, from examining the valence of thirst in mice, to studying courtship behavior in Drosophila, to restoring vision loss from retinitis pigmentosa in human trials^24–26^. Optogenetic channels work by absorbing photons and allowing specific types of ions to pass through the plasma membrane, activating or inhibiting the neuron. By introducing transgenic light-sensitive protein-coding genes into an organism, this technique can be used to activate or inhibit specific neurons that would otherwise be impossible to manipulate. Optogenetics provides advantages in temporal and spatial control, and a range of controls that enable experimental inference.

We recently described the first use of optogenetic tools to study mosquito behavior^8^. These tools rely on the red-light-activated cation channel CsChrimson, a red-shifted channelrhodopsin^27^. Microbial opsins, including CsChrimson, require a chemical cofactor, all-trans retinal, in order to absorb photons^28^. CsChrimson is fused to the tdTomato fluorescent reporter and is under the control of the QF2/QUAS binary expression system, enabling it to be flexibly combined with different driver lines^29^. To target CO_2_ sensory neurons, a driver line was used that expresses the QF2 transcription factor in neurons that express the Gr3 subunit of the CO_2_ receptor^30^. Mosquitoes showed minimal behavioral response after exposure to red light alone^8^; additionally, red light is most effective at penetrating biological tissue, making a red-light-activated opsin ideal for optogenetics^25^.

**Figure 1:**
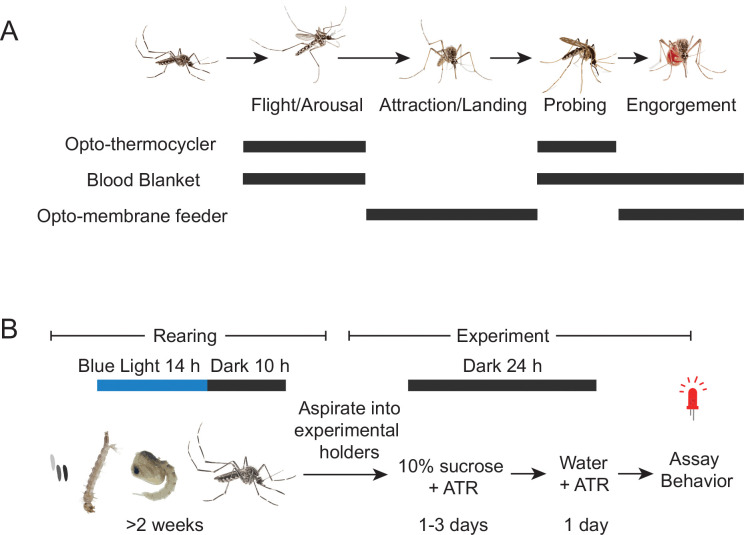
Assays and rearing for optogenetics in mosquitoes. **(A)** Schematic of mosquito host-seeking behavior. The steps of host seeking that are measured by each of the behavior assays are indicated with gray bars. **(B)** Timeline of rearing of mosquitoes for optogenetics experiments.

Here we describe protocols for three assays that measure different aspects of host-seeking and blood-feeding behavior combined with optogenetic stimulation (Figure 1). First, we provide a protocol for the rearing of optogenetic mosquito lines. The second section describes the opto-thermocycler, a modified PCR thermocycler that can be used to track arousal and probing when mosquitoes are exposed to temperature and light stimuli via light exposure. The third section describes the blood blanket assay, where mosquitoes are presented with constant access to a thin artificial blood meal on the base of a modified thermocycler, enabling tracking of flight, probing, and engorgement under exposure to varying light and changing temperature conditions. Finally, we describe the opto-membrane feeder, where mosquitoes are presented with a warm blood meal while in a canister, allowing tracking of both attraction to a blood source and engorgement under exposure to light stimuli. In addition to the expected outputs for each of these assays, machine vision models are described to track the body parts of the mosquitoes and identify different behaviors performed during the assay. The opto-thermocycler, blood blanket, and opto-membrane feeder assays offer ways to measure distinct stages of mosquito blood-feeding behavior while manipulating neural activity with light. These innovative techniques enable the study of mosquito neural circuits, which is critical for the development of solutions to a major public health threat.

## Protocol

### 1. Optogenetic Rearing

**1.1** Rear mosquitoes according to standard protocol^31^ with the following exceptions to maximize response of optogenetic lines during experimentation.

**1.2** Raise animals inside of a light-tight incubator set to 28°C and 80% humidity, on a 14 h blue light (450 nm), 10 h dark cycle (Figure 1B).

**NOTE:** The use of blue light minimizes activation of red-light activated channels, such as CsChrimson. All rearing steps occurring outside of the incubator should be performed in low light conditions.

**1.3** Begin experiments on adult mosquitoes 1–4 weeks post eclosion. Older mosquitoes may have stronger behavioral responses due to increased expression of CsChrimson.

**1.4** Two days prior to experimentation, gently aspirate female mosquitoes out of the cages and anesthetize at 4°C. Using a feather or aspirator, carefully transfer to the experimental containers according to the assay being used. Prepare a 0.2 M aliquot of all-*trans* retinal (ATR) in dimethyl sulfoxide (DMSO), stored at −20°C. Provide 10% sucrose and 400 μM ATR via soaked cotton wicks on the mesh of the container. Animals may feed for 1–3 days in the dark on this meal. NOTE: Feeding in the dark, as opposed to under blue light, prevents bleaching of the ATR (Figure 1B).

**1.5** One day before experimentation, replace the sucrose feeders with new feeders containing distilled water and 400 μM ATR. Animals remain in the dark until experimentation (Figure 1B).

**NOTE:** The experiments should be run during the mosquitoes’ morning or evening activity peaks to maximize response rate.

**1.6** Proceed to section 2 opto-thermocycler, section 3 blood blanket, or section 4 opto-membrane feeder, depending on which assay is to be used.

### 2. Opto-thermocycler

#### 2.1 Assembling components of the assay

**Figure 2:**
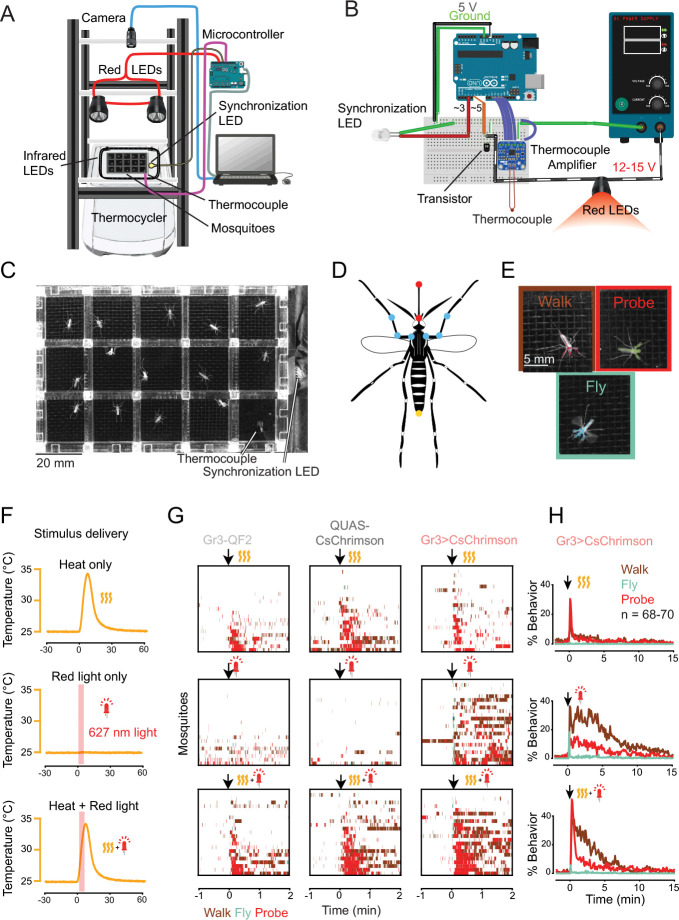
The opto-thermocycler quantifies arousal and search behaviors during optogenetic activation. **(A)** Schematic of opto-thermocycler assay consisting of a plate of mosquitoes on the block of a PCR thermocycler with overhead LEDs and a camera. **(B)** Circuit diagram for the microcontroller that detects the thermocycler temperature and controls the LEDs. **(C)** Still image from the assay depicting the acrylic plate that holds up to 14 mosquitoes. The thermocouple is in the bottom-right well. **(D)** Mosquito skeleton used in SLEAP model to estimate poses. Points labeled on the body include: the tip and base of the proboscis, used to detect probing behavior (shown in red); the front legs where they connect to the thorax, where the femur joins the tibia and where the tibia joins the tarsus (shown in blue); and the tip of the abdomen (shown in yellow). **(E)** Examples of mosquito walking, flying, and probing, with SLEAP predictions overlaid on top. **(F)** Output of stimulus delivery. Yellow wavy lines indicate body temperature heat stimuli and red LEDs indicate red light stimuli. **(G)** Ethograms of walking, flying, and probing behavior by Gr3-QF2, QUAS-CsChrimson, and Gr3>CsChrimson mosquitoes (n = 22–23) following stimulus delivery. **(H)** Long-lasting behavior following optogenetic activation in Gr3>CsChrimson mosquitoes (n = 68–70). Data in **(F-H)** are from a previous publication^8^.

**2.1.1** Make the acrylic plates to contain the mosquitoes (Figure 2C). See files in http://www.github.org/sorrellslab/opto-thermocycler for templates.

**2.1.1.1** To create the walls, laser cut a 3 mm (1/8 inch) acrylic sheet into four 10 cm by 1 cm rectangles and six 7 cm by 1 cm rectangles. Affix the rectangles with acrylic glue into a five-by-three matrix, where each unit is a 17 mm-by-17 mm-by-10 mm square. The template for these pieces is in the file 14_animal_thick_v3.ai.

**2.1.1.2** For the bottom of the chamber cut a 1.5 mm (1/16 inch) thick acrylic sheet to 6.985 cm (2 and 3/4 inch) by 10.16 cm (4 inch) with the bottom right well removed. Cut a 1.5 mm (1/16 inch) thick acrylic to a 6.985 cm (2 and 3/4 inch) inch by 10.16 cm (4 inch) rectangle for the lid. The template for these pieces is 14_animal_thin_v3.ai.

**2.1.1.3** Cut a UV-resistant black mesh sheet to the same size as the bottom acrylic piece (Figure 2C). Sandwich the mesh between the walls of the plate and the bottom piece, then glue them in place with acrylic glue.

**2.1.2** Construct a frame that will hold the camera and LEDs (Figure 2A).

**2.1.2.1** Build a frame with four pillars at each corner of the thermocycler using 60.96 cm (2 ft), 25 mm square optical construction rails. Use two 22.86 cm (9 inch), 25 mm construction rails to connect the front-right pillar to the back-right pillar and the front-left pillar to the back-left pillar, 30 cm from the bench. Connect the right and left pillars in the front with a 30.48 cm (1 ft), 25 mm construction rail, elevated 24 cm off the bench, and in the back with a 30.48 cm (1 ft), 25 mm construction rail elevated 42 cm off the bench.

**2.1.2.2** Attach 1-foot-long optical posts to the construction rail on the left and right sides of the frame. Tie six 627 nm (red) LEDs connected in series 24 cm above the PCR block of the thermocycler to each post. Tape a heat sink and lens on each LED to focus the light toward the block of the thermocycler.

**2.1.2.3** Fasten a 30.48 cm (1 ft) optical post to the top of the front two construction rail pillars. Use a right-angle clamp in the middle of the optical post to attach a 15.24 cm (6 inch) optical post facing inwards over the thermocycler block. Affix a camera with an infrared long-pass filter to the end so it is pointing down, 40 cm over the block.

**2.1.2.4** Using optical posts, create a frame that extends from the construction rail in a rectangle around the PCR block, elevated just above the surface. Affix a strip of infrared LEDs facing toward the PCR block for illumination of the mosquitoes at a right angle to the direction of the camera.

**2.1.2.5** Drape a blackout curtain over the structure to prevent interference from ambient light.

**2.1.3** Cover the surface and sides of the PCR block with a single layer of blackout tape to create a flat, even surface with a dark background.

**2.1.4** Place the thermocouple sensor on the PCR block in the lower right corner and secure it with blackout tape. Confirm even distribution of light across the PCR block using a power meter.

**2.1.5** On the edge of the PCR block, place an infrared LED to synchronize the video with the stimulus delivery output (Figure 2C). Cover the surface with black tape to allow the light to be detected without creating a glare in the camera.

**2.1.6** Wire the thermocouple, synchronization LED, and red light LEDs according to the diagram in Figure 2B.

**NOTE:** There are nine stimuli per experiment: three of heat only, three of light only, and three of heat and light simultaneously (Figure 2F).

**2.1.7** Download the required software (SpinView, CoolTerm, Arduino IDE).

**2.1.8** Download the Arduino code and thermocycler program from the GitHub repository http://www.github.com/sorrellslab/opto-thermocycler and transfer them to the computer and thermocycler, respectively. We provide programs to deliver red light, body-temperature heat, and the combination of the two stimuli. There are seven stimulus programs where the stimulus order is randomized between them. These can be modified to deliver different stimuli.

**NOTE**: The stimulus program detects changes in the surface temperature of the thermocycler via the thermocouple, then responds by synchronizing light delivery. Additional details can be found in comments within the stimulus programs.

### 2.2 Trial Preparation

**2.2.1** Rear mosquitoes according to the optogenetic rearing protocol; see section 1 and Figure 1B.

**2.2.2** Two days before the experiment, transfer 14 female mosquitoes with an aspirator to a separate container (i.e., a cage). Provide mosquitoes with a cotton wick or ball soaked in 10% sucrose + 400μM ATR (see step 1.4).

**NOTE:** Once mosquitoes receive ATR, they should be handled under dim light conditions.

**2.2.3** One day before the experiment, aspirate 14 female mosquitoes per acrylic plate and anesthetize them at 4°C. Carefully place one mosquito at a time into each of the wells with a feather or aspirator.

**2.2.4** Tape the side of the acrylic plate with Scotch tape to hold the lid in place. Do not fold the tape over the top or bottom of the acrylic plate, as it would obstruct the view of the camera.

**2.2.5** Place the acrylic plate on top of the cotton wicks soaked with 400μM ATR in distilled water (See step 1.5). Each of the 3 rows is aligned to a wick so all the mosquitoes have access to the water-ATR solution. Place the acrylic plate in a dark incubator overnight.

### 2.3 Running the trial

**2.3.1** Connect the camera, LEDs, and Arduino to the computer. Turn on the power supply. Wipe down the surface of the opto-thermocycler with 70% ethanol and allow it to dry.

**2.3.2** Using a dark container, transfer the acrylic plate of mosquitoes to the middle of the PCR block. Cover the optothermocycler with a blackout curtain.

**2.3.3** Open SpinView on the computer. Open the camera view under the **Cameras** tab. Plug in the infrared lights and make sure the PCR block and acrylic plate are visible in the camera view. If needed, adjust the acrylic plate so it is aligned, centered, and the thermocouple is visible in the bottom right corner of the acrylic plate. Ensure the synchronization light is in frame (Figure 2C).

**2.3.4** Adjust the settings on the camera and in SpinView to ensure a crisp, cropped video showing the acrylic plate. In the **Image Format** tab, adjust the **Width**, **Height**, **Offset X**, and **Offset Y** options to ensure the acrylic plate and synchronization light are visible. In the **Settings** tab, set **Acquisition Frame Rate Enable** to true and **Acquisition Frame Rate** to 30 fps. Turn **Exposure Auto** and **Gain Auto** off. Adjust the **Exposure Time** and **Gain** values to create a clean contrast between the black background and the mosquitoes.

**2.3.5** Click the red record button on the upper right side which opens a separate window to save the video file.

**2.3.6** In the pop-up window, set the file path at the top: Click **Browse** > Navigate to the experiment folder > Create a new folder with the trial name with the appropriate folder name.

**2.3.7** Set the number of frames to record to the appropriate number for the experiment (e.g., 43500) or to 0 to manually stop the recording.

**2.3.8** In the Videos tab, set the following fields: **Video Recording Type**: Mjpg, **Video File Split Size**: 1000 MB, **JPEG Compression Quality**: 65.

**2.3.9** Open the ‘TC_optothermo_lightheat_random1.x50prog’ program on the thermocycler and start it. The thermocycler should begin in a hold step at 25°C.

**2.3.10** Open the matching program ‘optothermo_lightheat_random1.ino’, on the Arduino IDE, and upload it to the board. Open the serial monitor and ensure that the temperature is approximately 25°C.

**2.3.11** Close the serial monitor in the Arduino IDE. Open CoolTerm to begin the acquisition of the Arduino serial monitor output. Make sure the serial port and channel match the serial port and channel on the Arduino IDE. The output should start showing on the CoolTerm window.

**2.3.12** Begin the recording of the Arduino output to a Text/Binary file on CoolTerm. Start the video recording on Spinview. Click **Resume** to end the hold on the thermocycler. Watch the Arduino output on CoolTerm to check if the temperature dip to 18°C was detected and the stimulus counter increased to 1, initiating the trial. Allow the assay to run until the trial is completed.

### 2.4 Clean up

**2.4.1** Stop the video recording and CoolTerm output acquisition. Close out of both programs. End the thermocycler program.

**NOTE:** This is recommended even when running another trial immediately after.

**2.4.2** Take the acrylic plate of mosquitoes from the thermocycler and place them in a −20°C freezer overnight to euthanize them.

**2.4.3** If you are starting another trial, repeat section 2.3.

**2.4.4** Unplug the camera and Arduino cables from the computer. Turn off the power supply. Unplug the infrared LEDs from the outlet.

**2.4.5** Clean acrylic plates by removing the Scotch tape and discarding the dead mosquitoes in biohazard waste. Spray the lid and acrylic plate with 70% ethanol and wipe all the sides of the wells and mesh with a gloved finger carefully. Rinse thoroughly with DI water and stack acrylic plates for storage with a paper towel separating lids to avoid scratching.

## 3. Blood Blanket Assay

### 3.1 Assembling components of the assay

**3.1.1** Construct the opto-thermocycler assay as described in section 2.1. The blood blanket assay will use the components of the opto-thermocycler with some modifications.

**Figure 3:**
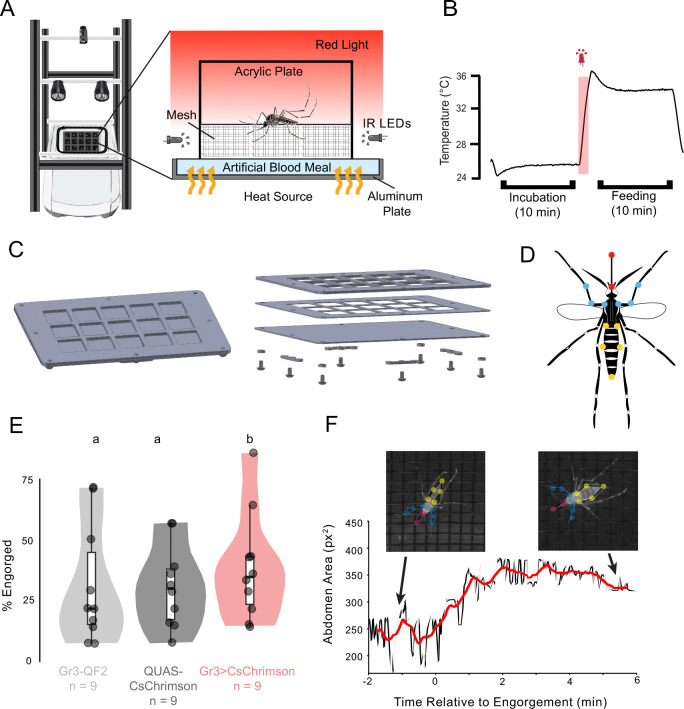
The blood blanket assay quantifies engorgement to optogenetic activation. **(A)** Schematic of the blood blanket assay. The mosquito is contained in an acrylic cage where accessible artificial blood is presented beneath a mesh screen. **(B)** Temperature plot with red light stimulus and expected state throughout the assay. **(C)** Schematic of aluminum blood blanket feeding plate assembly. **(D)** The SLEAP skeleton used for pose estimation included the points described in Figure 2D and in addition, the sides of the abdomen where they meet the thorax and the edges of the thickest part of the abdomen, to measure engorgement. **(E)** Violin plot depicting the mean percent engorged on the artificial blood meal per genotype. Significance was determined using the Kruskal-Wallis test, followed by a Dunn Test with Bonferroni correction. Different letters indicate a significance of p < 0.05 between groups. **(F)** Engorgement over time of a Gr3>CsChrimson animal as measured by abdomen area. The red trendline displays a 30-second sliding window over eight minutes of the trial, with x = 0 marking time when engorgement begins.

**3.1.2** Construct an aluminum blood meal feeding plate as seen in Figure 3C.

**3.1.2.1** Using a laser cutter or waterjet cutter construct the top and bottom of the plate. Construct the bottom of the plate by cutting an aluminum sheet 0.8 mm (1/32 inch) thick to 90 mm by 134 mm. Construct the top by cutting an aluminum sheet 1.6 mm (1/16 inch) thick to 90 mm by 134 mm. Cut 15 rectangular holes (17.2 mm by 18.6 mm) into the top plate.

**3.1.2.2** Sandwich between the aluminum parts a thin silicone gasket (0.5 mm thick) to prevent leakage. Cut using a laser cutter in the same shape as the aluminum top.

**3.1.2.3** Attach layers together by eight 0.47625 cm (3/16 inch), dome-topped hex 4–40 standard bolts. Use a forming tap to create threads for the bolts in the metal pieces. Connect these through round and “W” washers on the bottom of the plate for added security when placed atop the thermocycler.

**3.1.3** Download the Arduino code and thermocycler program for the blood blanket assay from the GitHub repository https://github.com/sorrellslab/bloodblanket and transfer them to the computer and thermocycler, respectively.

**3.1.4** Prepare stock solutions.

**3.1.4.1** Prepare aliquots of 20 mM ATP in 25 mM NaHCO_3_ and store at −20°C until use.

**NOTE:** Prepare a fresh ATP solution every six months. Avoid repeated freeze-thaw cycles.

**3.1.4.2** Prepare 1 M NaHCO_3_ and 5M NaCl (store each at room temperature).

### 3.2 Experiment Preparation

**3.2.1** Rear mosquitoes and transfer to acrylic plates as described in sections 1 and 2.2.

**3.2.2** The day of the experiment, prepare a 10 mL minimal artificial blood meal (110 mM NaCl, 20 mM NaHCO_3_, and 1.5 mM ATP) by adding 750 μL 20 mM ATP, 220 μL of 5 M NaCl, and 200 μL of 1 M NaHCO_3_ stock solutions to 8.83 mL deionized water. Mix thoroughly by inverting.

**NOTE:** Make the artificial blood meal fresh prior to each experiment.

**3.2.3** Set up SpinView recording according to steps 2.3.4 and 2.3.5.

**3.2.4** Open the ‘Red-light-Only__BB_.ino’ Arduino file (or desired stimulus file) on the Arduino IDE.

### 3.3 Running Trials

**3.3.1** Pipette 650 μL of the minimal artificial blood meal to each well of the plate, ensuring that the meal entirely fills each well.

**3.3.2** Stretch a 10.16 cm (4 inch) x 10.16 cm (4 inch) square of parafilm over a 20.32 cm (8 inch) diameter ring. Place stretched parafilm over the plate, pressing on the edges until parafilm sticks to the plate. Use a scalpel to cut around the plate to remove excess parafilm.

**3.3.3** Place the plate directly on top of the PCR block to allow maximum heat transfer. Ensure that the plate is firmly in contact with the block as shown in Figure 3A.

**NOTE:** Blackout tape is used on the surface of the PCR block for the opto-thermocycler but not for the blood blanket.

**3.3.4** Place the thermocouple onto the surface of the parafilm in the lower right corner of the aluminum plate. Connect the camera, LEDs, and Arduino to the computer. Turn on the power supply.

**3.3.5** Start the ‘BB_thermocycler_program.x50prog’ program on the thermocycler.

**3.3.6** Using a dark container and dim room lights to minimize light exposure, transfer the acrylic plate of mosquitoes onto the aluminum plate, ensuring each well is aligned with the mosquitoes. Ensure the thermocouple is in the right corner of the plate and is in contact with the parafilm to measure the temperature of the artificial blood meal.

**3.3.7** After a 10-minute acclimation period, begin the trial. In the Arduino IDE, upload the ‘Red-light-Only__BB_.ino’ program with the arrow button. Close the serial monitor. In CoolTerm, press **Connect** to see the current temperature. Using this starting temperature, adjust the trigger temperature in the Arduino script to be approximately 0.6°C below the starting temperature.

**3.3.8** In the CoolTerm window, select **Capture to Text/Binary file** and start data collection.

**3.3.9** Start video recording in the record window in SpinView.

**3.3.10** Click **Resume** to end the hold on the thermocycler. Watch the Arduino output on CoolTerm to ensure that the heat dip was detected and the trial has started.

**3.3.11** After the duration of the trial (10 minutes in this case), click **Stop Recording** on SpinView. In CoolTerm, stop the recording.

**3.3.12** Remove the plate of mosquitoes from the blood blanket plate and move them to the cold room to count the number of engorged mosquitoes.

**3.3.13** Between trials, clean the surface of the PCR block by wiping the surface with 70% ethanol. Remove the parafilm and rinse the aluminum plate with deionized water.

**3.3.14** Repeat section 3.3 for further trials.

### 3.4 Clean up

**3.4.1** Stop recording all programs and quit. Unplug USB cords for the camera, Arduino, and infrared lights. Shut down the computer. Stop the ‘thermocycler_program.x50prog’ program running on the thermocycler.

**3.4.2** Place mosquito plates in a −20°C freezer overnight to kill the mosquitoes.

**3.4.3** Rinse the aluminum plate with deionized water. Wipe the acrylic plate with 70% ethanol, removing any debris and residue with gloved fingers from the wells, then rinse with deionized water and leave to dry overnight before loading more mosquitoes.

**NOTE**: Plates should be dried overnight after washing to make sure there is no moisture left before loading mosquitoes. If there is moisture this will cause condensation on the lid and will interfere with the video.

## 4. Opto-membrane Feeder Assay

### 4.1 Assembling components of the assay

**Figure 4:**
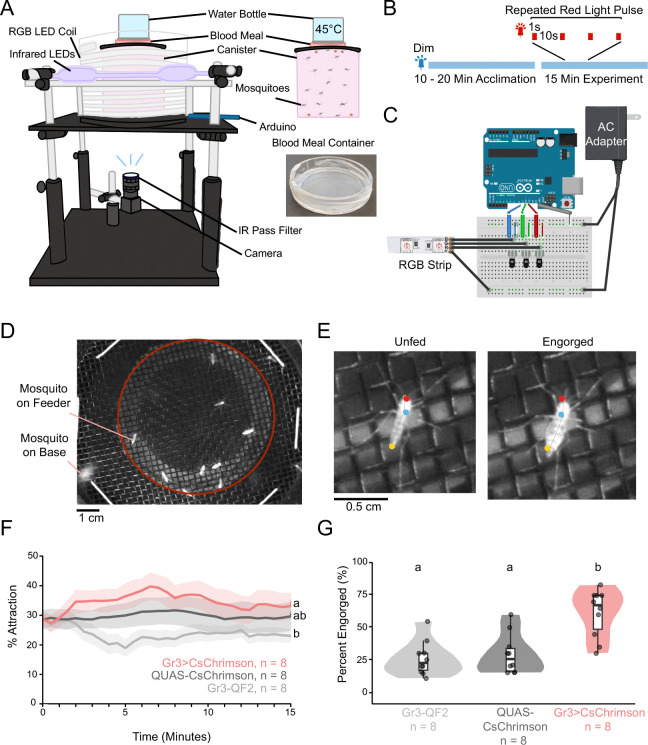
The opto-membrane feeder assay measures attraction and blood feeding. **(A)** Schematic of the opto-membrane feeder with individual components. **(B)** Timeline of experiment trials. The blue line represents dim blue light throughout the acclimation period and experiment. A segment of the experimental stimulus is shown, representing 1 s pulses of red light spaced 10 s apart. **(C)** Diagram depicting the wiring for the opto-membrane feeder. **(D)** Example frame from the opto-membrane feeder video. Mosquitoes in the red circle are counted to determine percent attraction. **(E)** Examples of an unfed mosquito and an engorged mosquito, with the head, thorax, and abdomen labeled by the SLEAP tracking model for the opto-membrane feeder. **(F)** Line graph depicting mean percent occupancy on the blood meal region over time, by genotype. Mean is plotted every 30 s. Shaded regions depict standard error. **(G)** Violin plot depicting the mean percent engorged on a blood meal by genotype. In **(F, G)**, Significance determined by the Kruskal-Wallis test, followed by the Dunn Test with Holm correction. Different letters indicate significant differences between groups.

**4.1.1** Assemble a frame using optomechanical components and a 30.48 cm (12 inch) x 30.48 cm (12 inch), black, 0.635 cm (1/4 inch) thick, acrylic platform. Create a hole in the center of the acrylic with a diameter of 11.43 cm (4.5 inch) using a laser cutter. Cut holes 2.54 cm (1 inch) from each of the corners, to allow the acrylic to rest on the supports of the frame, approximately 23.495 cm (9.25 inch) above the base. In the center, attach a black acrylic ring with a 19.05 cm (7.5 inch) inner diameter and a 20.32 cm (8 inch) outer diameter (Figure 4A). Templates for all acrylic components are in the GitHub repository https://github.com/sorrellslab/opto-membranefeeder along with the Arduino programs.

**NOTE**: The specific materials used to assemble this platform are not critical, so more cost-effective options may be available.

**4.1.2** Attach a camera with an infrared long-pass filter below the center hole, pointing up toward the mosquito canister. Adjust the aperture to be fully open, allowing selective focus on the mesh top of the canister (Figure 4A).

**4.1.3** Construct canisters to contain the mosquitoes (Figure 4A).

**4.1.3.1** Cut a clear, polycarbonate tube with a diameter of 11.43 cm (4.5 inch) into 12.7 cm (5 inch) cylindrical segments.

**4.1.3.2** Build bottoms for the canisters by laser cutting circles with a 11.43 cm (4.5 inch) diameter from 0.3175 cm (1/8 inch) thick acrylic. The edges of the circles are etched with the laser so that they are inset into the tube. Attach to the tubes with plastic epoxy.

**4.1.3.3** Create an inset lid using black 0.635 cm (1/4 inch) and 0.3175 cm (1/8 inch) acrylic and UV-resistant black mesh. The inner ring has a diameter of 10.795 cm (4.25 inch), while the outer ring has a diameter of 12.065 cm (4.75 inch), with the black mesh stretched over the smaller ring. The mesh should be sewn on through laser cut holes with waxed thread.

**NOTE:** Canisters should always be placed on paper towels to avoid scratching the acrylic bottoms through which video is recorded.

**4.1.4** Construct acrylic containers for blood meal delivery (Figure 4A).

**4.1.4.1** Cut a ring with a 5.08 cm (2 inch) inner diameter and 6.6 cm (2.6 inch) outer diameter from 0.15875 cm (1/16 inch) clear acrylic.

**4.1.4.2** Attach this to another clear ring with a 5.842 cm (2.3 inch) inner diameter and 6.604 cm (2.6 inch) outer diameter using acrylic glue.

**4.1.5** Place a metal mesh cylinder with a 13.97 cm (5.5 inch) height and a 19.05 cm (7.5 inch) diameter inside the black acrylic ring at the center of the platform.

**4.1.6** Inside the metal mesh, attach a coil of RGB LED lights spaced 3.81 cm (1.5 inch) from the exterior of the canister, to be controlled by an Arduino Uno board which rests on the black acrylic platform (Figure 4C).

**4.1.7** Outside the metal mesh, attach a ring of 850 nm infrared LEDs to the frame using zip ties, facing inward.

**4.1.8** Plug a USB cord into the Arduino Uno board and connect to the computer that will be used to run the software. Connect a USB cord from the camera to the computer. Turn the connection to the internet off on the computer.

**4.1.9** Place the opto-membrane structure in a dark incubator or environmental room at 26°C and 80% relative humidity for the duration of the experiments.

### 4.2 Preparing the mosquito canisters

**4.2.1** Prior to the experiment, wash mosquito canisters by spraying 70% ethanol and wiping gently with a soft sponge. Rinse the canisters with deionized water and air-dry overnight.

**4.2.2** Two days prior to the experiment, sex mosquitoes under cold anesthesia and place 20 females into each canister. Provide access to ATR according to steps 1.4 and 1.5.

**4.2.3** At this time, genotypes should be blinded to the experimenter. The order of the trials should be rotated between days to minimize differences in behavior outcomes due to experimental timing.

### 4.3 Running Trials

**4.3.1** Prepare the blood meal.

**4.3.1.1** Place 119 mL (4 oz) bottles of water into a 45°C heat bath, one for each trial.

**4.3.1.2** Prepare 5 mL aliquots of defibrinated sheep blood. Store at 4°C and invert prior to use to prevent separation.

**4.3.1.3** Place first blood aliquot into a 45°C heat bath for at least 20 minutes prior to the trial.

**4.3.1.4** Thaw ATP on ice.

**4.3.1.5** Stretch a 5.08 cm (2 inch) x 5.08 cm (2 inch) square of parafilm over the acrylic lid on the side with the larger, flat circle, creating a well for the blood meal (see Figure 4A).

**4.3.2** Wipe the computer with 70% EtOH to remove human odors. Turn on the computer and connect it to an external hard drive. Connect the camera and the Arduino to the computer. Plug the RGB and infrared lights into a power source.

**4.3.3** Open the SpinView software and the Arduino file for the appropriate stimulus.

**4.3.4** Create a folder on the external hard drive to contain the experiment data.

**4.3.5** Upload the blue_only Arduino file to provide dim blue 471 nm light from the RGB LEDs to the arena (following Figure 4B).

**4.3.6** Without exposing mosquitoes to excess CO_2_ from breath, examine the canister for the trial and record any deaths prior to the experiment.

**4.3.7** Place the canister on the center of the platform and allow acclimation under dim blue light for 10 to 20 minutes prior to stimulus exposure.

**NOTE**: The assay is run under dim blue light because complete darkness inhibits mosquito flight.

**4.3.8** Enable video capture.

**4.3.8.1** In the SpinView program, select the camera. In the **Settings** tab, adjust the exposure and gain as needed to maintain high contrast between the light-colored insects and the dark background (Figure 4D).

**4.3.8.2** Click the red record button on the top bar to adjust video capture settings.

**4.3.8.3** Set the file path by clicking **Browse** and navigating to the external hard drive.

**4.3.8.4** Create a new folder with a descriptive name for each trial. Do not include spaces in the folder name.

**4.3.8.5** In the **Save Options** tab, select **Capture 0 frames**, which will allow video to record until manually stopped. Under **Recording Mode**, select **Buffered**. In the **Videos** tab, set the following fields: **Video Recording Type**: Mjpg, **Video File Split Size**: 1000 MB, and **JPEG Compression Quality**: 65.

**4.3.9** Immediately before stimulus exposure, pipette 500 μL of 20 mM ATP, for a final concentration of 2 mM ATP, into the prepared 5 mL blood aliquot. Invert the tube several times to mix.

**4.3.10** Pour the ATP-blood solution into the blood meal container.

**4.3.11** On top of the blood meal, place an inverted 119 mL (4 oz) bottle filled with 45°C water to maintain the temperature of the blood close to human body temperature throughout the experiment.

**4.3.12** In the SpinView software, select **Start Recording**.

**4.3.13** Without stimulating mosquitoes with your breath, place the blood meal on top of the mesh lid of the canister so mosquitoes can pierce the parafilm membrane through the mesh.

**4.3.14** Upload the Arduino program for ‘1sON_10sOFF.ino’ (Figure 4B), or the desired stimulus.

**4.3.15** Place the blood aliquot into the heat bath for the following trial.

**4.3.16** After the trial has run for 15 minutes, select **Stop Recording**.

**4.3.17** Upload the ‘blue_only.ino’ stimulus file to the Arduino board to stop the red light stimulus.

**4.3.18** Remove the blood meal holder and water bottle. Remove the canister and anesthetize the mosquitoes at 4°C.

**4.3.19** To perform additional trials, return to section 4.3.

**4.3.20** Visually examine the anesthetized mosquitoes to determine and record the number that engorged on blood by looking for a blood-filled abdomen.

### 4.4 Clean up

**4.4.1** Close all programs.

**4.4.2** Eject external hard drive and disconnect from the computer.

**4.4.3** Shut down the computer.

**4.4.4** Unplug all cords connected to the computer and to power sources.

**4.4.5** Clean the mosquito canisters as previously described in step 4.2.1.

**4.4.6** Clean acrylic blood meal holders.

**4.4.6.1** Fill a half-sized polycarbonate pan 2/3 full with water and add 100 mL of bleach.

**4.4.6.2** Add acrylic blood meal holders and plastic bottles to the pan. Cover with a lid and let soak for 10 minutes.

**4.4.6.3** Rinse bleach and remove parafilm.

**4.4.6.4** Scrub gently and rinse to remove any blood remnants.

## 5. Analysis of behavior

**5.1** Prepare the videos for analysis by compressing them into .mp4 files using ffmpeg. If your videos are split, merge them into one video file using any video editing software.

**5.2** Open the training videos in SLEAP in grayscale. If the video recording settings are appropriate, everything should be within frame and moving mosquitoes should not be blurry.

**NOTE:** Other body part tracking software can be used. The SLEAP version used in this paper is 1.4.1. Refer to SLEAP documentation for up-to-date protocols.

**5.3** Create a skeleton that labels the relevant parts of the animals according to the behaviors being assessed (Figure 2D, 3D and 4E).

**5.4** Using the “Random frame” option under the **Suggested frame** tab, randomly pick 10 frames and label each mosquito as an instance. If certain parts of the mosquito are obstructed (i.e. the proboscis tip is inserted into the mesh during probing), label your best guess for the location of the body part. Train a model on the labeled frames with these settings:

**5.4.1** For a Top-Down multi-animal model (Used for the opto-thermocycler and blood blanket model): In the **Training Pipeline** tab, set **Max Instances** to 14. In the **Centroid Model Configuration** tab, set **Crop Size** to Auto, set **Plateau Min. Delta** to 1e-04, set **Plateau Patience** to 10, set **Stride** to 8, and the **Anchor Part** as thorax.

**5.4.2** For a Bottom-Up multi-animal model (Used for the opto-membrane feeder): In the **Training Pipeline** tab, set **Max Instances** to no max. In the **Bottom-Up Model Configuration** tab, set **Crop Size** to Auto, set **Plateau Min. Delta** to 1e-04, set **Plateau Patience** to 10, and set **Stride** to 16.

**5.5** Predict on a set amount of frames with these settings: In the **Inference Pipeline** tab, for the opto-thermocycler and blood blanket, set **Max Instances** to 14, **Tracker** to simple**, Max number of tracks** to 14, and **Connect Single Track Breaks** to True. For the opto-membrane feeder, set No Max for **Max Instances** and **Max number of tracks**, **Tracker** to simple, and **Connect Single Track Breaks** to True.

**5.6** Correct the predicted frames and re-train. Iterate through cycles of prediction, labeling, and training until the model predicts at a high accuracy and precision. This can take up to 150 labeled frames, depending on the number of edge cases in your videos. Use the model on experimental videos and export into .csv or .h5 for behavior-tracking analysis.

## Results

Mosquitoes were reared under blue light-dark cycle (14:10 LD; Figure 1B) to synchronize circadian rhythms so experiments can be run during wake periods. Two days before experiments, mosquitoes were transferred to total darkness and provided with the opsin cofactor ATR. If mosquitoes are asynchronous or not fed ATR, they will show low activity in behavioral assays. Which behavior assay to use depends on the steps of host seeking and blood feeding that are of interest as shown in Figure 1A. The genotypes used for each assay were Gr3>CsChrimson and two genetic controls: the driver line Gr3-QF2 and the effector line QUAS-CsChrimson.

The opto-thermocycler assay (Section 2) allows for the assessment of mosquito arousal and probing behavior over long periods of time in response to heat and light stimuli. The use of a thermocycler allows for quick surface heating and cooling that is synchronized with optogenetic activation via a thermocouple and microcontroller (Figure 2A, B). The three mosquito genotypes were placed in acrylic plates with isolated wells with mesh bottoms in which they demonstrate walking, flying, and probing behavior (Figure 2C). The position of mosquito body parts was determined using pose-tracking algorithms, such as SLEAP^32^ and DeepLabCUT^33^ (Figure 2D, E). Behavior classification algorithms can be used starting from pose tracking (e.g. SimBA^34^, A-SOiD^35^) or video (e.g. FERAL^36^) input. Figure 2E shows the SLEAP output during different host-seeking and feeding behaviors, which can be classified by the velocity of the thorax and the distance between the base and tip of the proboscis, respectively. Here, we show the output of behavior tracking using Animal Part Tracker and JAABA^37^. Mosquitoes were exposed to either a heat increase, a light stimulus, or both heat and light stimuli simultaneously, then a break of 20 minutes before the next stimulus^8^. (Figure 2F). When exposed to heat, all three genotypes exhibit a short period of probing (Figure 2G). Importantly, when exposed to a 5-second light stimulus, the Gr3>CsChrimson mosquitoes become aroused or “activated,” showing increased walking and probing behavior that lasts up to 10–15 minutes (Figure 2G, 2H). The opto-thermocycler allows for precise optogenetic and heat stimulation, individually or in combination, to multiple individual mosquitoes at once, making it a useful assay to study arousal and probing behavior.

The blood blanket assay (Section 3) is built upon the opto-thermocycler assay but provides a palatable artificial blood meal so mosquito engorgement can be quantified (Figure 3A). The three genotypes (n = 9) were each presented with an artificial blood meal in an aluminum feeding plate (Figure 3C). A 5-second pulse of red light (~627 nm) was administered to optogenetically activate CO_2_ sensory neurons and the thin layer of artificial blood meal was heated from 25°C to 35°C (as measured at the surface by a thermocouple; Figure 3B). The control mosquito lines, Gr3-QF2 and QUAS-CsChrimson, demonstrated a similar average engorgement (Figure 3E, p = .586). In contrast, the Gr3>CsChrimson line demonstrated significantly higher rates of feeding, with an average engorgement rate of 43% (Figure 3E, p = .040 versus Gr3-QF2 and p = .132 versus QUAS-CsChrimson). To track engorgement of the mosquitoes throughout the experiment, SLEAP was used to track points on the body of the mosquito. This was done by creating a skeleton that included 5 nodes on the abdomen of the mosquito (Figure 3D) and measuring the change in the abdominal area (Figure 3F). These results show that the blood blanket assay can be used to assess the effects of optogenetic activation of neuronal populations on engorgement of mosquitoes on an artificial blood meal.

The opto-membrane feeder assay (Section 4), where mosquitoes are presented with access to a warm blood meal in a cylindrical arena, allows for study of mosquito attraction to a blood source and engorgement. To determine if optogenetic activation of CO_2_ sensory neurons is sufficient to cause attraction to and feeding on a blood source, Gr3>CsChrimson and control mosquitoes (n = 8 for each genotype) were presented with access to a warm blood meal in the opto-membrane feeder under exposure to a repeated 1 second pulse of bright red light every 10 seconds for a duration of 15 minutes (Figure 4B). To measure attraction to the membrane feeder, a SLEAP model with three points was used to track the head, thorax, and abdomen of landed mosquitoes, counting those within the region of interest (Figure 4D, Figure 4E). Gr3>CsChrimson mosquitoes showed increased occupancy on the blood meal region over time, significantly higher than the Gr3-QF2 control (Figure 4F, p = .027). Although the Gr3>CsChrimson mosquitoes consistently showed higher attraction than the QUAS-CsChrimson control beyond the 1-minute point, this difference was not significant, potentially due to a relatively small sample size (Figure 4F, p = .310). The control genotypes Gr3-QF2 and QUAS-CsChrimson showed similar engorgement of 26% and 29% respectively (Figure 4G, p = .351). In contrast, Gr3>CsChrimson mosquitoes demonstrated a significantly higher percentage of engorgement than either control, at an average rate of 61% (Figure 4G, p < .001 versus Gr3-QF2 and p = .002 versus QUAS-CsChrimson). These results indicate that the opto-membrane feeder assay is a viable means to assess the effects of optogenetic activation of CO_2_ neurons on mosquito attraction to and engorgement on a blood meal.

## Discussion

Here, we have described detailed protocols for assaying the sufficiency of neuronal cell types to drive steps in host attraction and biting. Biting behavior consists of a series of behavioral steps from long-range detection to short-range attraction, piercing of skin, and engorgement. In principle, neuronal types could control specific actions or steps, bias behavior toward specific sequences, increase the duration of the whole behavior, or play many other roles. Thus, the multiple assays we present are necessary to dissect the function of neurons in this process.

Optogenetic tools have been created that are sensitive to wavelengths from blue (~450 nm) to red (650 nm), spanning the visible spectrum. Red light is particularly useful in insects as they typically have low sensitivity and/or behavioral responses to these wavelengths^38^. Interestingly, mosquitoes show attraction during flight to patches of visual stimuli with long wavelengths present in human skin tones^39^. However, in our assays control mosquitoes showed minimal responses to broadly applied red light, even when very bright^8^ (Figures 2–4). We recommend genetic controls lacking either the driver or effector constructs and no-light controls to ensure behavioral effects are interpreted accurately. A control lacking the rhodopsin cofactor ATR is often used for optogenetics experiments in other species; however, the fish food used to feed mosquito larvae contains vitamin A, the precursor to ATR, so this is a less effective control in this system.

Optogenetic stimuli that closely recapitulate natural neuronal activity patterns are most likely to elicit the behavior of interest. Therefore, if neural recordings are available, the stimulus can be designed to match the observed intensity and temporal dynamics. When this is not known, the function of neurons can be screened using different stimuli and behavioral contexts, with the caveat that the results may not reflect the function of neurons in naturalistic behavior. Despite this, driving activity of neurons out of the natural range can be used to probe circuit or behavioral properties in unique ways just as electrophysiology can be used to probe cellular properties.

Currently, optogenetic tools have only been used in sensory neurons in the mosquito, an application that provides some advantages over the delivery of the natural stimulus. Traditional strategies of measuring mosquito host seeking rely on the delivery of real CO_2_ gas. Using real CO_2_ requires air flow that has been filtered and humidified, which requires complex experimental setups to be able to precisely deliver and remove in short time increments^9,10,40,41^. Air flow and humidity are both cues for mosquito attraction and navigation, making optogenetic delivery appealing as a method to study the effects of olfactory stimuli separately from these other stimuli.

A major advantage of optogenetics in the mosquito will be realized when it is combined with drivers that express in interneurons that cannot be directly stimulated by any other approach. Newly emerged resources such as the *Aedes aegypti* Mosquito Cell Atlas open the door for new advances in the field of mosquito neurogenetics^42^. Future work will be needed to develop such drivers for probing mosquito neural circuits. Similarly, additional optogenetic tools for inhibition will be needed to determine whether a neuron type is necessary for a particular behavior. Although here we demonstrate assays for host attraction and blood feeding, these approaches could be extended to study additional steps of host seeking and behaviors such as mating, oviposition, and nectar feeding. These tools will provide new insight into how the behavior of the mosquito is controlled at the neural level and contribute to new approaches to prevent the spread of mosquito-borne diseases.

### Materials

**Table:** 

Name of Material	Equipment Company	Catalog Number	Comments/Description
AC Adapter	Jameco ReliaPro	2197581	Opto-membrane feeder structure
Acrylic Glue	United States Plastic Corp	97562	Opto-membrane feeder canister, blood blanket cage
Adenosine 5’-triphosphate disodium salt hydrate	Sigma Aldrich	A6419-1G	Artificial blood meal
All Trans-Retinal	Spectrum Chemical	R3041-1GM	Optogenetic rearing
Aluminum Breadboard	Thorlabs	MB12	Opto-membrane feeder structure
Aluminum Sheet 0.0625 in	McMaster-Carr	89015K37	Blood blanket feeding plate
Aluminum Sheet 0.03125 in	McMaster-Carr	89015K11	Blood blanket feeding plate
Arduino Uno Rev3 Microcontroller Board	Arduino	A000066	Opto-membrane feeder programming
Blackout drape	Set Shop	6520SF	Opto-thermocycler and blood blanket
Black Fiberglass Window Screen	Breakthrough Premium Products	IHLRS3684BL	Blood blanket cage
Black Tape	Thorlabs	T137-2.0	Opto-thermocycler
Black Zip Ties	Cable Ties And More	ct242	Opto-membrane feeder structure
Blue LED Lights	21LEDUSA	RB5730BLWP	Mosquito rearing
Bottle	SKS Bottle & Packaging	0604-07	Opto-membrane feeder blood heater
Bottom Feeder Pellets	API	5352983	Feeding larvae
Camera	Blackfly	U3-13S2M-CS, FLI	Recording video for assays
Click Counter	ULine	H-7350	Thinning larvae
Concentrated Bleach (8.25% Sodium Hypochlorite)	VWR	89501-620	Opto-membrane feeder cleaning
Computer	Asus	X1407Q	Running Assays
Defibrinated Sheep Blood	Hemostat Laboratories	DSB100	Artificial blood meal
Dome-topped hex 4-40 bolt	McMaster-Carr	92949A105	Blood blanket feeding plate
Epoxy	Loctite	1363118	Opto-thermocycler acrylic plate, opto-membrane feeder structure
Ethanol	Decon Labs	2716	Sanitization
External Hard Drive	LaCie	LAC9000298	Video Storage
Four Inch Cotton Wick	Richmond Dental	201226	Feeding adult mosquitoes
Gr3-QF2 Mosquitoes	N/A	N/A	Genetic reagent, DOI: 10.1016/j.cell.2013.12.044
¼ Inch Black Acrylic	McMaster-Carr	8560K259	Opto-membrane feeder structure
⅛ Inch Black Acrylic	McMaster-Carr	8650K32	Opto-membrane feeder structure
⅛ Inch Clear Acrylic	McMaster-Carr	8536K134	Opto-membrane feeder canister
Incubator with Opaque Door	Caron	7340-25	Mosquito rearing
Infrared LED Light	Adafruit	387	Opto-thermocycler and blood blanket
Infrared LED Strip	Waveform Lighting	7031.85	Opto-thermocycler, blood blanket, and opto-membrane structure
Infrared Pass Filter	Edmund Optics	65-796	Recording video for assays
Insect Cage	Bugdorm	DP1000	Adult mosquito enclosure
Large Soup Cup	WebstaurantStore	50016SOUPPLA	Mosquito rearing
LED Light	Luxeon Star	SP-01-D9	Red light stimulus for opto-thermocycler and blood blanket
LED lens	Luxeon Star	10209	Opto-thermocycler and blood blanket
LED lens holder	Luxeon Star	10235	Opto-thermocycler and blood blanket
LED lens tape	Luxeon Star	LT-01	Opto-thermocycler and blood blanket
LED thermal tape	Luxeon Star	LXT-S-12	Opto-thermocycler and blood blanket
LED heat sink	Luxeon Star	N25-15B	Opto-thermocycler and blood blanket
Optical Construction Rails	Thorlabs	XE25L12, XE25L24, XE25L09, XE25T4, RA90, TRA6, TR12	Opto-thermocycler and blood blanket structure
Optical Post	Thorlabs	TR12	Opto-membrane feeder structure
Paper Towels	Pacific Blue	27385	Canister cleaning
Parafilm	Amcor	PM-996	Blood blanket membrane, opto-membrane meal container
PCR thermocycler	Eppendorf	2231001196	Opto-thermocycler and blood blanket
Plastic Portion Cup	Choice	999P2C	Feeding adult mosquitoes
Plastic Portion Cup Lid	Choice	999PL2	Feeding adult mosquitoes
Polycarbonate Pan	Cambro	21410CWCHCL	Rearing larvae
Polycarbonate Pan Lid	Cambro	10CWCH135	Rearing larvae
Polycarbonate Pan Half Sized	Cambro	24CW135	Opto-membrane meal cleaning
Polycarbonate Pan Half Sized Lid	Cambro	20CWCH135	Opto-membrane meal cleaning
Polycarbonate Tube	McMaster-Carr	8585K56	Opto-membrane feeder canister
Power Meter	Coherent	1299161	All assays
QUAS-CsChrimson Mosquitoes	N/A	N/A	Genetic reagent, DOI: 10.7554/eLife.76663
RGB Lights	Digikey	289-1189-ND	Opto-membrane feeder stimulus delivery
Right Angle Clamp	Thorlabs	RA90	Opto-membrane feeder structure
Silicone Gasket, 0.5 mm	McMaster-Carr	1460N22	Blood blanket feeding plate
Sodium Bicarbonate, 1M buffer soln., pH 8.0	Thermo scientific	J62495.AP	Artificial blood meal
Sodium Chloride solution	Sigma-Aldrich	S5150-1L 100 3532552	Artificial blood meal
Spray Bottle	Uline	S-11686	Sanitization
Sucrose	Sigma Aldrich	S0389-500G	Feeding adult mosquitoes
Scotch Magic Tape	3M	104	Opto-thermocycler and blood blanket acrylic lid
Thermocouple (Type T)	Harold G Schaevitz Industries LLC	CPTC-120-X-N	Opto-thermocycler and blood blanket
Thermocouple amplifier	Adafruit	MAX31856	Opto-thermocycler and blood blanket
Timer	EnduraLight	RAC-MT	Controlling light cycle for mosquito rearing
Transfer Pipette	Thermo Scientific	SAM-225	Thinning larvae
Trash Bag	Uline	S-23040	Insect transportation
UV Resistant Mesh	McMaster-Carr	87655K13	Opto-membrane feeder canister
Water	Sigma Aldrich	W4502-1L	Artificial blood meal for blood blanket
Waxed Thread	McMaster-Carr	6356K91	Opto-membrane canister lid
Wire Mesh	McMaster-Carr	9322T65	Opto-membrane feeder structure
